# Supplementary material for: Predictors of Frequent Emergency Department Use and Hospitalization among Patients with Substance-Related Disorders Recruited in Addiction Treatment Centers
Source: Int J Environ Res Public Health. 2022 May 28;19(11):6607. doi: 10.3390/ijerph19116607 (PMC9180458; doi:10.3390/ijerph19116607)
Supplement: Supplementary file 1 [file ijerph-19-06607-s001.zip › ijerph-1702568-supplementary.pdf]

Table S1. Codes for substance-related disorders, mental disorders and chronic physical illnesses according to the International Classification of Diseases, Ninth or Tenth revisions

Diagnoses	*International Classification of Diseases, Ninth Revision (ICD-9)*	*International Classification of Diseases, Tenth Revision, Canada (ICD-10-CA)*
**Substance-related disorders (SRD)[a, b]**		
Alcohol-related disorders	3030*, 3039*, 3050* (alcohol abuse or dependence); 2910*, 2918* (alcohol withdrawal), 2911*-2915*, 2919*, 3575, 4255, 5353, 5710-5713 (alcohol-induced disorders); 9800, 9801, 9808, 9809 (alcohol intoxication)	F101*, F102* (alcohol abuse or dependence); F103, F104* (alcohol withdrawal); F105-F109, K700*-K704*, K709*, G621*, I426, K292*, K852, K860, E244, G312, G721, O354 (alcohol-induced disorders); F100*, T510, T511*, T518, T519 (alcohol intoxication)
Cannabis-related disorders	3043, 3052 (cannabis abuse or dependence)	F121, F122 (cannabis abuse or dependence); F123-F129 (cannabis-induced disorders); F120, T407 (cannabis intoxication)
Drugs-related disorders other than cannabis	3040-3042, 3044-3049, 3053-3057, 3059 (drug abuse or dependence); 292.0 (drug withdrawal); 2921, 2922, 2928, 2929 (drug-induced disorders); 9650, 9658, 9670, 9676, 9678, 9679, 9694-9699, 9708, 9820, 9828 (drug intoxication)	F111, F131, F141, F151, F161, F181, F191, F112, F132, F142, F152, F162, F182, F192 (drug abuse or dependence); F113-F114, F133-F134, F143-F144, F153-F154, F163-F164, F183-F184, F193-F194 (drug withdrawal) F115-F119, F135-F139, F145-F149, F155-F159, F165-F169, F185-F189, F195-F199 (drug-induced disorders); F110, F130, F140, F150, F160, F180, F190, T400-T406, T408, T409, T423, T424, T426, T427, T435, T436, T438, T439, T509, T528, T529 (drug intoxication)
**Mental disorders (MD)[a]**		
**Serious MD**		
Schizophrenia spectrum and other psychotic disorders	295* (schizophrenic disorders); 297* (paranoid states); 298* (other nonorganic psychoses)	F20* (schizophrenic disorders); F22* (persistent delusional disorders); F23 (acute and transient psychotic disorders); F24* (induced delusional disorder); F25* (schizoaffective disorders); F28* (other psychotic disorder not due to a substance or known physiological condition); F29* (unspecified psychosis not due to a substance or known physiological condition); F448 (other dissociative and conversion disorders); F481 (depersonalization - derealization syndrome)
Bipolar disorders	2960-2966 (manic disorders); 2968 (other affective psychoses); 2969 (unspecified affective psychoses)	F300-F302, F308, F309 (manic episode); F310-F317, F318, F319 (bipolar episode)
**Personality disorders**	3010 (paranoid personality disorder); 3011 (affective personality disorder); 3012 (schizoid disorder); 3013, 3014 (obsessive-compulsive personality disorder); 3015 (histrionic personality disorder); 3016 (dependent personality disorder); 3017 (antisocial	F600 (paranoid personality disorder); F61 (mixed and other personality disorders); F340 (cyclothymic disorder); F341 (dysthymic disorder); F601 (schizoid personality); F603 (borderline personality disorder); F605 (obsessive-compulsive personality disorder); F604 (histrionic personality disorder); F607 (dependent personality disorder); F602

	personality disorder); 3018 (other personality disorders); 3019 (unspecified personality disorder)	(antisocial personality disorder); F609 (unspecified personality disorder); F21 (schizotypal personality); F606 (avoidant personality disorder); F608 (other specified personality disorders); F681 (factitious disorder); F688 (other specified disorders of adult personality and behavior); F69 (unspecified disorder of adult personality and behavior)
**Common MD**		
Anxiety disorders	300 (except 3004); 3000 (anxiety states); 3002 (phobic anxiety disorders); 3003 (obsessive-compulsive disorder); 3001 (hysteria); 3006 (other anxiety disorder); 313 (disturbance of emotions specific to childhood and adolescence)	F40 (phobic anxiety disorders); F41(other anxiety disorders); F42 (obsessive-compulsive disorder); F45 (somatoform disorders); F48 (other neurotic disorders); F93, F94 (disturbance of emotions specific to childhood and adolescence)
Depressive disorders	3004 (neurotic depression)*; 311, 3119* (depressive disorder, not classified elsewhere)	F320- F323 (major depressive disorder, single episode); F328 (other depressive episodes); F329 (depressive episode, unspecified); F330-F334 (major depressive disorder, recurrent); F338 (other recurrent depressive disorders); F339 (recurrent depressive disorder, unspecified); F348 (other persistent mood [affective] disorders); F380, F381 (persistent mood [affective] disorder, unspecified); F388 (other specified mood [affective] disorders); F39 (unspecified mood [affective] disorders); F412* (mixed anxiety and depressive disorder)*
Adjustment disorders	3090 (brief depressive reaction); 3092 (adjustment reaction with predominant disturbance of other emotions, include: abnormal separation anxiety); 3093 (adjustment reaction with predominant disturbance of conduct); 3094 (adjustment reaction with predominant disturbance of other emotions and conduct); 3098 (other specified adjustment reactions); 3099 (unspecified adjustment reaction)	F430 (acute stress reaction); F431 (post-traumatic stress disorder); F432 (adjustment disorders); F438 (other reactions to severe stress); F439 (reaction to severe stress, unspecified)
Attention deficit/hyperactivity disorder	314	F900; F901; F908; F909
**Others MD**	2930, 2931 (transient organic psychotic conditions); 2940, 2941 (other organic psychotic conditions); 2990, 2991*, 2998, 2999 (pervasive developmental disorders); 290, 2941, 3310, 3312 (dementia); 3020-3029 (sexual deviations and disorders); 3070-3079 (special symptoms or syndromes, not elsewhere classified include anorexia nervosa, tics); 312	F060-F069 (other mental disorders due to known physiological condition); F840, F841, F842, F843, F844, F845 (pervasive developmental disorders); F00x-F03, F051, G30, G311(dementia); F500-F502 (eating disorders); F520-F529 (sexual dysfunction, not caused by organic disorder or disease); F510-F515 (nonorganic sleep disorders); F950-F952, F958, F959 (tic disorders); F980-F986, F988, F989 (other behavioral and emotional disorders with onset usually occurring in childhood and adolescence); F630-F633, F638, F639 (habit and impulse disorders); F70-73, F78, F79 (mental retardation)

	(disturbance of conduct, not elsewhere classified); 3150-3159 (specific delays in development); 316 (psychic factors associated with diseases classified elsewhere); 317-318 (mental retardation)	
**Suicide attempt**[a, c]		X60-Y09, Y870, Y871, Y35-Y36, Y890, Y891
**Chronic physical illnesses**[d]		
Renal failure	4030, 4031, 4039, 4040, 4041, 4049, 585, 586, 5880, V420, V451, V56	I120, I131, N18, N19, N250, Z49, Z940, Z992
Cerebrovascular illnesses	430-438	G45, G46, I60-I69
Neurological illnesses	3319, 3320, 3321, 3334, 3335, 3339, 334–335, 3362, 340, 341, 345, 3481, 3483, 7803, 7843	G10–G12, G13, G20, G21–G22, G254, G255, G312, G318, G319, G32, G35, G36, G37, G40, G41, G931, G934, R470, R56
Endocrine illnesses (hypothyroidism, fluid electrolyte disorders and obesity)	2409, 243, 244, 2461, 2468; 2536, 276; 2780	E00, E01, E02, E03, E890; E222, E86, E87; E66
Any tumor without or with metastasis (solid tumor without metastasis; lymphoma)	140-172, 174, 175, 179-195, 196–199; 200, 201, 202, 2030, 2386, 2733	C00–C26, C30–C34, C37–C41, C43, C45-C58, C60–C76, C77-C79, C80; C81-C85, C88, C900, C902, C96
Chronic pulmonary illnesses	490–505, 5064, 5081, 5088	I278, I279, J40-J47, J60-J64, J65, J66, J67, J684, J701, J703
Diabetes complicated and uncomplicated	2500-2502, 2503; 2504-2509	E102-E108, E112-E118, E132-E138, E142-E148; E100, E101, E109, E110, E111, E119, E130, E131, E139, E140, E141, E149
Cardiovascular illnesses (congestive heart failure, cardiac arrhythmias, peripheral vascular illnesses, valvular illnesses, myocardial infarction, hypertension) and pulmonary circulation illnesses	4021, 4041, 428; 4260, 4267, 4269,4270–4274,4276–4279, 7850, V450, V533; 394–397, 424,7463–7466, V422, V433; 093, 440, 441, 4431– 4439, 4471, 5571, 5579, V434; 4109, 4129; 4010, 4011, 4019, 4020, 4021, 4029, 4050, 405,4051, 4059, 4372; 4150, 4151, 416; 4170, 4178, 4179	I099, I110, I130, I132, I255, I420, I425–I429, I43, I50, P290; I441–I443, I456, I459, I47–I49, R000, R001, R008, T821, Z450, Z950; A520, I70-I72, I730, I731, I738, I739, I771, I790, K551, K558, K559, Z958, Z959; I05–I08, I091, I098, I34–I39, Q230–Q233, Q238, Q239, Z952, Z953, Z954I210-I214, I219, I220, I221, I228, I229, I252; I101, I100, I11, I1500, I1501, I1510, I1511, I1521, I1581, I1590, I1591, I674; I26, I27, I280, I288, I289
Other chronic physical illness categories (blood loss anemia, ulcer illnesses, liver illnesses, AIDS/HIV, rheumatoid arthritis/collagen vascular illnesses, coagulopathy, weight loss, paralysis, deficiency anemia)	2800, 2809; 286, 2871, 2873-2875; 5317, 5319, 5327, 5329, 5337, 5339, 5347, 5349; 0702, 0703, 0704, 0705, 4560–4562, 5723, 5728, 5733, 5734, 5739, V427; 042–044; 1361, 446; 7010, 7100–7104, 7105, 7108, 7109, 7112, 714, 7193, 720, 725, 7285, 7288, 7293; 260–263, 7832, 7994; 3341, 342, 343, 3440-3446, 3448, 3449; 2801, 2809, 281, 2859	D500; K257, K259, K267, K269, K277, K279, K287, K289; B20-B24; D65–D68, D691, D693-D696; B18, I85, I864, I982, K700- K703, K709 K711, K713–K715, K716, K717, K721, K729, K73, K74, K754, K760, K761, K763, K764, K765, K766, K768, K769, Z944; L900, L940, L941, L943, M05, M06, M08, M120, M123, M30, M31, M32–M35, M45, M460, M461, M468, M469; G041, G114, G80, G81, G82, G83; E40–E46, R634, R64, D51–D53, D63, D649; D501, D508; D509

[a] All diagnoses identified in MED-ECHO (*Maintenance et exploitation des données pour l'étude de la clientèle hospitalière*, hospitalization database) prior to 2005-06 (financial year: April 1 to March 31), or in RAMQ (*Régie de l'assurance maladie du Québec,* Quebec Health Insurance Plan database) for the full study period were based on the International Classification of Diseases Ninth Revision (ICD-9), which included a 4-digit code. The Canadian Tenth Revision (ICD-10-CA) was used in MED-ECHO in 2006-07+ and in BDCU (Banque de données communes des urgencies, emergency department (ED) database). Diagnoses related to all the above databases were considered, and all data integrated each year, for each patient. MED-ECHO is the only database that includes several diagnoses: principal diagnosis and numerous secondary diagnoses. In the databases used in this study, MD were considered only as principal diagnoses, but SRD as both principal and secondary diagnoses, considering that SRD are often underdiagnosed. See **Figure 1** for the construction of each variable integrated into the study and specific related databases.

[b] SRD diagnostic codes were based on the RAMQ, MED-ECHO or BDCU databases. These diagnoses were also identified in the SIC-SRD (*Système d'information clientèle pour les services de réadaptation dépendances*) based on standardized questionnaires available from 2009-10 to 2015-16, i.e., the adapted Quebec version of the Addiction Severity Index (IGT: *Indice de gravité d'une toxicomanie*) or the Global Appraisal of Individual Needs (GAIN). The research team had final scores only, not the raw data, for these standardized instruments administered by clinicians.

[c] Diagnostic codes for suicide attempts were registered in the MED-ECHO (hospitalization) database. ED use for reasons of suicide ideation or attempt was reported by ED triage nurses and registered in the BDCU database (ED database); as they are not diagnostic codes, they were not reported in this table.

[d] The list of chronic physical illnesses is based on an adapted and validated version of the Elixhauser Comorbidity Index, integrating the Charlson Index, which consists of 32 major categories of physical illnesses (see reference in the Method section). In this list of chronic physical illnesses, three categories of MD and two of SRD (identified with an asterisk (*)) were also included in the list of MD-SRD, thus appearing twice.
